# The Role of Resilience in Relation to Depression, Anxiety, Stress, and Post-Traumatic Stress Symptoms Among Syrian Refugee Adolescents in Türkiye

**DOI:** 10.3390/children13091183

**Published:** 2026-09-02

**Authors:** Ahmet Özaslan, Murat Yıldırım, Fatma Nur Öztürk, İrem Aktar Songür, Muhammed Hakan Aksu, Esra Güney, Ahmet Tanhan, Feten Fekih-Romdhane, Carlos Laranjeira, Juan Gómez-Salgado

**Affiliations:** 1 Child and Adolescent Psychiatry Department, Gazi University, Ankara 06560, Türkiye; ahmetozaslan@gazi.edu.tr (A.Ö.);; 2 Child Protection Research and Application Center, Gazi University, Ankara 06560, Türkiye; 3 Psychology Research Centre, Khazar University, Baku AZ1096, Azerbaijan; 4 Department of Psychology, Faculty of Science and Letters, Agri Ibrahim Cecen University, Ağrı 04100, Türkiye; 5 Department of Psychology, Faculty of Arts and Sciences, Haliç University, İstanbul 34060, Türkiye; 6 Psychiatry Department, Gazi University, Ankara 06560, Türkiye; 7 Department of Counseling, Adıyaman University, Adıyaman 02040, Türkiye; 8 Faculty of Medicine of Tunis, Tunis El Manar University, Tunis 1007, Tunisia; 9 The Tunisian Center of Early Intervention in Psychosis, Department of Psychiatry “Ibn Omrane”, Razi Hospital, Manouba 2010, Tunisia; 10 School of Health Sciences, Polytechnic University of Leiria, Campus 2, Morro do Lena, Alto do Vieiro, Apartado 4137, 2411-901 Leiria, Portugal; 11 Centre for Innovative Care and Health Technology (CiTechCare), Polytechnic University of Leiria, Campus 5, Rua Das Olhalvas, 2414-016 Leiria, Portugal; 12 Comprehensive Health Research Centre (CHRC), University of Évora, 7000-801 Évora, Portugal; 13 Department of Medicine and Public Health, Faculty of Labour Sciences, University of Huelva, 21071 Huelva, Spain; 14 Safety and Health Postgraduate Program, Universidad Espíritu Santo, Guayaquil 092301, Ecuador

**Keywords:** resilience, depression, anxiety, stress, post-traumatic stress symptoms, refugee adolescents

## Abstract

**Highlights:**

**What are the main findings?**

- Resilience was significantly and negatively associated with depression, anxiety, stress, and post-traumatic stress symptoms (intrusion and avoidance) among Syrian refugee adolescents in Türkiye.
- Resilience explained a small but significant amount of unique variance (1–3%) in these outcomes, beyond age and gender.

**What are the implications of the main findings?**

- Higher resilience was associated with fewer depression, anxiety, stress, and post-traumatic stress symptoms.
- Refugee adolescents rarely reach specialist mental health services, which makes schools a practical setting for support.

**Abstract:**

**Objectives:** Mental health problems and post-traumatic stress symptoms are prevalent among adolescent refugees, yet little is known about the role of resilience in relation to these difficulties among Syrian adolescents. This study examined the unique contribution of resilience to mental health problems and post-traumatic stress symptoms after controlling for age and gender. **Methods:** Participants were 501 Syrian refugee adolescents residing in Ankara, Türkiye (63.67% male; aged 12–18 years, M = 15.72, SD = 1.61), recruited through multistage cluster sampling in 15 schools. They completed the Brief Resilience Scale, the Children’s Revised Impact of Event Scale (CRIES), the Depression, Anxiety, and Stress Scale, and a socio-demographic questionnaire. Five hierarchical multiple regressions were conducted, entering age and gender at Step 1 and resilience at Step 2. **Results:** After controlling for age and gender, resilience was significantly and negatively associated with depression (β = −0.11, *p* < 0.05), anxiety (β = −0.19, *p* < 0.01), stress (β = −0.15, *p* < 0.01), intrusion (β = −0.14, *p* < 0.01), and avoidance (β = −0.15, *p* < 0.01), explaining a modest but consistent proportion of unique variance (1–3%). **Conclusions:** These findings suggest that resilience is a modest but consistent correlate of mental health and post-traumatic stress symptoms among Syrian refugee adolescents in Türkiye. Longitudinal studies incorporating trauma exposure and multi-level resilience resources are needed before causal or intervention-related conclusions can be drawn.

## 1. Introduction

In recent years, forced migration and displacement have reached unprecedented levels worldwide. By mid-2025, an estimated 117.3 million people were forcibly displaced, and children under 18 years of age make up around 40% of the displaced population [1]. Exposure to war-related violence, the hardships of displacement, and the challenges of resettlement can have profound effects on refugee youth during critical stages of development, thereby increasing their vulnerability to mental health problems [2]. An estimated 22.7% of child and adolescent refugees meet diagnostic criteria for post-traumatic stress disorder (PTSD), 13.8% for depression, and 15.8% for anxiety disorders [3]. These estimates are considerably higher than World Health Organization estimates for the general adolescent population worldwide, in which anxiety disorders affect an estimated 4.1% of 10–14-year-olds and 5.3% of 15–19-year-olds, and depression affects 1.3% and 3.4%, respectively [4]. Türkiye hosts one of the largest refugee populations in the world. The number of Syrians under temporary protection peaked at 3,737,369 in 2021 and has since declined, following the return movements that began after the political transition in Syria in December 2024, to 2,224,593 as of 20 August 2026, of whom 1,057,700 (47.5%) are under 18 years of age [5]. Studies of Syrian adolescents in Türkiye have reported prevalence ranges from 18.3% to 61.3% for probable PTSD, from 27.0% to 51.5% for depression, and from 29.5% to 72.8% for anxiety [6,7,8,9], and Syrian refugee adolescents report significantly more symptoms than their Turkish peers [10]. This elevated risk has been linked to a range of pre- and post-migration adversities [2,11,12]. Among these outcomes, post-traumatic stress is one of the most prominent, and studies using the Children’s Revised Impact of Event Scale (CRIES) have documented high levels of post-traumatic stress symptoms among Syrian refugee adolescents [8,13].

Post-traumatic stress disorder (PTSD) is a trauma- and stressor-related disorder that follows exposure to actual or threatened death, serious injury, or sexual violence, whether experienced directly, witnessed, or learned about in relation to a close family member or friend [14]. Beyond this exposure criterion, the diagnosis requires the persistence of re-experiencing, avoidance, negative alterations in cognition and mood, and hyperarousal symptoms for more than one month, together with clinically significant distress or functional impairment [14]. Exposure to a potentially traumatic event is therefore a necessary but not sufficient condition for PTSD, and, as noted above, roughly one in five child and adolescent refugees meet diagnostic criteria [3]. Among Syrian refugee children and adolescents in Türkiye, the most frequently reported war-related experiences include hearing or witnessing explosions and gun battles, seeing dead or wounded people, the violent death of someone important, and mistreatment or torture [6,7]. Among these, the death of an important person has been independently associated with probable PTSD [6]. Across the wider literature, exposure to war-related trauma is the pre-migration risk factor with the strongest evidence base, whereas post-migration stressors such as discrimination, acculturative stress, economic hardship, and lack of food or shelter in resettlement appear to compound this risk further [2,7,11,12].

These symptoms comprise two core clusters: intrusion (e.g., involuntary memories and nightmares) and avoidance of trauma-related reminders. Both have been supported as distinct factors in Syrian refugee adolescents [15]. The two clusters also tend to reinforce one another, since avoidance impedes the processing of traumatic memories and thereby sustains intrusive re-experiencing [16]. Despite all these difficulties, a substantial proportion of refugee adolescents can adapt well. In a large multi-country European sample, nearly half of them showed a profile of consistently low symptoms, including low post-traumatic stress [17], and in one study conducted in Türkiye, 60.7% of Syrian adolescents reported moderate to exceptionally high levels of resilience [9]. Resilience, known as the capacity for positive adaptation in the face of significant adversity, or the ability to bounce back from stress, has therefore been proposed as a key protective factor among conflict-affected youth [18,19,20].

How resilience operates in refugee adolescents, however, remains poorly understood. Current models describe resilience not as a fixed trait but as a dynamic process arising from interacting resources across multiple levels of the child’s social ecology [20,21]. A recent scoping review of 49 studies mapped these resources at the individual (adaptive coping, self-esteem, optimism), family (cohesion, supportive parenting, stable routines), and community levels (social support, school and community participation) [22]. These resources have been linked to better mental health outcomes among refugee children facing trauma and displacement-related stress [23]. Cognitive models propose that intrusion and avoidance are maintained when fragmented trauma memories and avoidant coping prevent the processing and contextual integration of the traumatic experience, sustaining a sense of ongoing threat [16]. Resilience has been proposed to interrupt this cycle: more resilient individuals may appraise stressors as less threatening and rely more on active and less on avoidant coping, which in turn has been associated with fewer post-traumatic stress symptoms over time [24]. A similar protective pattern has been observed for other outcomes: among Syrian refugee children in Jordan, higher resilience was associated with lower depressive symptoms, and trauma exposure was no longer significantly related to depression among the most resilient children [25]. However, much of this evidence—including the reviews and multi-country studies cited above—comes from high-income resettlement settings [17,23], whereas refugee youth living in neighbouring host countries and low- and middle-income settings remain underrepresented in the literature [2,26]. Moreover, resilience has rarely been a specific focus of this research. A recent systematic review of studies on refugee children in Nordic countries found that none explicitly examined it [27]. Even where resilience has been studied, the focus has typically been on its correlates rather than on whether it explains unique variance in mental health outcomes beyond age and gender [9]. It therefore remains unclear whether resilience is independently associated with both general mental health problems (depression, anxiety, and stress) and trauma-specific post-traumatic stress symptoms (intrusion and avoidance) among Syrian refugee adolescents in Türkiye.

The present study therefore aimed to address this gap by examining the unique role of resilience in relation to symptoms of depression, anxiety, stress, intrusion, and avoidance, while controlling for age and gender. We hypothesised that, even after accounting for age and gender, resilience would be significantly and negatively associated with symptoms of depression, anxiety, stress, intrusion, and avoidance.

## 2. Materials and Methods

### 2.1. Participants

The study sample comprised 501 Syrian refugee adolescents residing in Türkiye and attending either secondary or high school. The data were obtained from a larger study investigating the mental health of Syrian refugee adolescents. While previous publications from this project have examined different research questions (e.g., Özaslan et al. [28]), the present study focuses specifically on the role of resilience in relation to depression, anxiety, stress, and post-traumatic stress symptoms. Of the 557 adolescents invited across the selected classes, 22 (3.9%) declined to participate, yielding 535 in-class participants (participation rate: 96.1%). An additional 21 adolescents who could not be reached in class during periods of pandemic-related remote schooling subsequently completed the same measures online, for a total participant pool of 556. Of these, 55 were excluded from the present analyses owing to missing data on the study measures, yielding the final analytic sample of 501. The previously published report from the same project [28] analysed 488 in-class participants with complete data on the measures examined in that study (the DASS-21, the General Help-Seeking Questionnaire, and the Self-Stigma of Seeking Psychological Help Scale); the two analytic samples therefore constitute overlapping subsets of the same participant pool, with differences in sample size reflecting measure-specific complete-case availability and the inclusion of the online participants in the present study. In total, 480 participants were included in both analytic samples; the remaining 21 participants in the present sample were the online respondents, and 8 participants from the earlier report were excluded here owing to missing data on the resilience or CRIES-8 measures. Of the participants, 63.67% were boys and 36.33% were girls, with a mean age of 15.72 years (SD = 1.61). Grade-level distribution varied, with only a small proportion of participants in grades 5 and 6 (0.80% and 1.60%, respectively), whereas the largest proportions were in grades 9 and 10 (28.34% and 21.56%, respectively). Further demographic information is presented in Table 1.

### 2.2. Measures

The Depression Anxiety Stress Scale-21 (DASS-21) was used to assess symptoms of depression, anxiety, and stress during the previous week. The instrument comprises 21 self-report items distributed equally across three seven-item subscales. Items are rated from 0 (did not apply to me at all) to 3 (applied to me very much or most of the time), and the relevant items were summed to obtain separate depression, anxiety, and stress scores ranging from 0 to 21, with higher scores indicating greater symptom severity. The raw subscale sums were retained rather than multiplied by two because the scores were analysed as continuous variables and were not interpreted using the normative severity categories of the 42-item scale. Sariçam [29] demonstrated sufficient reliability and validity for the Turkish version of the scale, and Ali et al. [30] confirmed the reliability and validity of the Arabic version. In the present sample, Cronbach’s alpha coefficients were 0.730 for depression, 0.733 for anxiety, and 0.719 for stress.

The Brief Resilience Scale (BRS) was used to assess participants’ capacity to recover from challenging situations [18]. The scale consists of six self-reported items, each rated on a 5-point Likert scale ranging from 1 (strongly disagree) to 5 (strongly agree). Following the scoring procedure of the Turkish adaptation [31], a total score (range: 6–30) was computed by summing all items after reverse-coding items 2, 4, and 6, with higher scores indicating greater resilience. As the summed score is a linear transformation of the item-mean score used in the original scale [18], the two scoring approaches are statistically equivalent and yield identical standardised coefficients. The BRS has demonstrated adequate psychometric properties in both Arabic [32] and Turkish [31] versions. Cronbach’s alpha in the present sample was 0.402.

The Children’s Revised Impact of Event Scale-8 (CRIES-8) is an adaptation of the Impact of Event Scale, originally developed to assess post-traumatic stress reactions. It evaluates two core symptom clusters—intrusion and avoidance—focusing on re-experiencing the traumatic event and avoidance of trauma-related stimuli and emotions [33]. Designed as a child-friendly instrument, the CRIES-8 is widely used in research involving children at risk following traumatic events, and its Turkish version has demonstrated satisfactory psychometric properties [34]. In the present study, the CRIES-8 was used to assess intrusion and avoidance symptom levels. The eight items are rated on a four-point frequency scale; four items assess intrusion (items 1, 3, 6, and 7; possible range: 4–16) and four assess avoidance (items 2, 4, 5, and 8; possible range: 4–16), with higher scores indicating more severe symptoms. In the present sample, Cronbach’s alpha coefficients were 0.837 for intrusion and 0.770 for avoidance. The CRIES-8 was administered with its standard instructions, which ask respondents to indicate how frequently each statement applied to them during the past week in relation to a stressful event they have experienced. No specific index event (e.g., war- or displacement-related trauma) was designated; participants therefore completed the scale with reference to a self-identified stressful experience.

### 2.3. Procedure

Participants were recruited using a multistage cluster sampling procedure, in which districts, then schools, then classes were successively selected, and all students in the selected classes were invited to participate [35]. Within the education policies of the Ministry of National Education, refugee and local adolescents are educated together in mixed classes. In Ankara, where the study was conducted, the Directorate of National Education granted permission to conduct the study in 15 secondary or high schools. Secondary-school classes typically comprised approximately 25 students and high-school classes around 30 students. To ensure representation from areas with a high concentration of Syrian refugee adolescents, five secondary schools and ten high schools were randomly selected from those registered with the Ankara Directorate of National Education in the Altındağ, Mamak, and Keçiören districts, where Syrian refugee adolescents comprised roughly one-third of the students in each class. Within each selected school, five classes were then randomly selected across all grade levels (e.g., 9AB, 10BB, 11CS).

Detailed information on the purpose of the study, the administration of the scales, and the scientific use of the data was provided by the researchers in both Turkish and Arabic in the presence of an interpreter. The self-report scales, in Arabic or Turkish according to the adolescents’ preference, were administered in approximately one class hour (45 min). Written informed consent was obtained from parents or legal representatives, and written assent from all participating adolescents, prior to in-class administration. The majority of participants completed the paper-and-pencil forms in class; during periods of pandemic-related remote schooling, 21 participants who could not be reached in class completed the same measures online after providing electronic informed consent. Of the 501 participants, 41 (8.2%) completed the Arabic versions of the self-report measures, and 460 (91.8%) completed the Turkish versions.

### 2.4. Statistical Analysis

To assess bivariate relationships, Pearson correlation coefficients were calculated. The dataset did not exhibit violations of regression assumptions, such as multicollinearity, linearity, homoscedasticity, or multivariate normality. Five hierarchical multiple regression analyses were performed to examine the association of resilience with mental health problems and post-traumatic stress symptoms while controlling for demographic variables, entering age and gender at Step 1 and resilience at Step 2. All analyses were conducted using IBM SPSS Statistics version 27.0 (IBM Corp., Armonk, NY, USA), with a significance level set at *p* < 0.05.

## 3. Results

The preliminary analysis (see Table 2) indicated that skewness values ranged from −0.44 to 0.48 and kurtosis values from −1.04 to 1.00, suggesting a relatively normal distribution of the study variables. Table 2 also presents the bivariate correlations. Resilience demonstrated a significant negative correlation with depression, anxiety, stress, intrusion, and avoidance. All mental health and post-traumatic stress symptoms were positively correlated with one another. Age showed significant positive correlations with anxiety, intrusion, and avoidance, whereas gender was positively correlated with resilience but negatively correlated with all other variables.

Five independent hierarchical multiple regression analyses were conducted, with age and gender entered at Step 1 and resilience at Step 2 (Table 3). In the final model for depression, resilience was negatively associated with symptoms (B = −0.135, β = −0.108, 95% CI −0.244 to −0.026, *p* = 0.016) and explained an additional 1.1% of the variance (ΔR^2^ = 0.011, ΔF[1, 497] = 5.88, *p* = 0.016). Resilience was also negatively associated with anxiety (B = −0.218, β = −0.186, 95% CI −0.320 to −0.117, *p* < 0.001; ΔR^2^ = 0.034, ΔF[1, 497] = 17.89), stress (B = −0.183, β = −0.145, 95% CI −0.293 to −0.073, *p* = 0.001; ΔR^2^ = 0.021, ΔF[1, 497] = 10.63), intrusion (B = −0.142, β = −0.142, 95% CI −0.228 to −0.055, *p* = 0.001; ΔR^2^ = 0.020, ΔF[1, 497] = 10.29), and avoidance (B = −0.143, β = −0.145, 95% CI −0.227 to −0.058, *p* = 0.001; ΔR^2^ = 0.021, ΔF[1, 497] = 11.04). Thus, after adjustment for age and gender, resilience accounted for a modest but consistent proportion of unique variance across all five outcomes.

## 4. Discussion

The present study examined the unique contribution of resilience to symptoms of depression, anxiety, stress, intrusion, and avoidance in a large school-based sample of Syrian refugee adolescents in Türkiye, after controlling for age and gender. As hypothesised, higher resilience was significantly and negatively associated with all five outcomes, indicating that resilience was related to fewer symptoms across both general mental health problems and trauma-specific post-traumatic stress reactions in this population.

These findings are consistent with a growing body of research linking resilience to better mental health among conflict-affected and refugee youth. Among Syrian refugee children in Jordan, higher resilience was associated with lower depressive symptoms [25], and in Lebanon, refugee children with greater environmental resources were more likely to belong to a low-symptom profile [26]. In Türkiye specifically, resilience has been found to correlate negatively with depression, anxiety, and post-traumatic stress symptoms among Syrian adolescents [9]. Notably, protective resources may also moderate the impact of trauma in war-affected adolescents; for example, prosocial behaviour buffered the effect of trauma on post-traumatic stress severity among adolescents in Gaza [36]. The present cross-sectional design, however, could not test such a buffering role. Our findings extend this literature by showing that resilience was associated with both general (DASS-21) and trauma-specific (CRIES-8) symptoms within the same sample, suggesting that it may be relevant across multiple symptom domains rather than to a single disorder.

The association between resilience and symptoms was strongest for anxiety and weakest for depression. One possible interpretation is that the capacity to recover from stress assessed by the Brief Resilience Scale is conceptually closer to arousal-related symptoms such as anxiety and stress than to the anhedonic features of depression [18]. With respect to demographic factors, girls reported significantly fewer symptoms than boys across all five outcomes. This pattern runs counter to evidence that internalising and post-traumatic stress symptoms are often more common among refugee girls [2,17] and to a population-based study of Syrian refugee children in Istanbul, in which girls showed a higher prevalence of common mental disorders than boys [13]. This may reflect unmeasured differences in trauma exposure between girls and boys, which have been reported in other Syrian samples in Türkiye [7], and the predominantly male composition of the present sample may also have contributed. An earlier study of Syrian refugee adolescents in Türkiye found that age and gender were not significantly associated with CRIES-8 symptoms [8], whereas in the present sample gender was associated with both intrusion and avoidance; this difference may reflect variation in sample size and composition. In bivariate analyses, older age was also associated with higher anxiety and avoidance symptoms, although these associations fell just short of conventional significance in the final adjusted models (*p* = 0.052 and *p* = 0.062, respectively).

The present design is consistent with a compensatory model, in which resilience relates directly to fewer symptoms; it does not test a protective (buffering) model, in which resilience would weaken the effect of trauma exposure. The negative association with intrusion and avoidance is consistent with cognitive models of post-traumatic stress [16]: more resilient adolescents may appraise stressors as less threatening and rely less on avoidant coping, allowing better processing of traumatic memories and, in turn, fewer post-traumatic stress symptoms [24]. Within a social–ecological framework, the Brief Resilience Scale captures resilience primarily at the individual level, whereas family- and community-level resources, such as family cohesion and social support, were not assessed [21,22]. Had these broader resources been measured, they might have accounted for variation in adolescents’ mental health that the individual-level resilience score alone could not capture.

The unique variance explained by resilience was modest, ranging from 1% to 3% across outcomes. Several considerations place this in context. First, the associations were consistent in direction across all five outcomes, although the outcomes were intercorrelated and the effects were small. Second, age and gender together explained a comparable proportion of variance (2–5%), so the contribution of resilience was similar in magnitude to that of these established demographic correlates. Third, the brief, unidimensional resilience measure may have attenuated the observed associations through restricted construct coverage and measurement error [37]. Conversely, the exclusive reliance on self-report raises the possibility of common-method variance, which would be expected to inflate rather than attenuate associations among measures completed by the same informant; the net effect of these opposing biases on the true magnitude of the associations is therefore uncertain.

These findings also have tentative practical relevance. Refugee youth tend to underuse outpatient mental health services and often present late or in crisis [38], and self-stigma has been shown to fully mediate the relationship between symptoms and help-seeking among Syrian refugee adolescents in Türkiye [28]. Together, these findings suggest that many adolescents may not independently seek clinical care and that preventive efforts should reach them where they already are. Schools offer such a setting, and strength-based, school- and community-based programmes that target modifiable resources have been recommended for war-affected children [22,39]. In practical terms, school-based screening that captures both general and trauma-specific symptoms could help identify adolescents in need of referral, and low self-reported resilience may serve as one additional marker of vulnerability within such screening. Classroom-based psychosocial programmes delivered by trained teachers or lay counsellors already target the coping and social-support resources that resilience frameworks emphasise [22,39]. The present findings do not establish that adding resilience-focused components to such programmes would reduce symptoms; they indicate only that resilience is a consistent, if modest, correlate of symptoms and therefore a reasonable candidate target for prospective evaluation. More broadly, contemporary public mental health frameworks increasingly situate resilience in crisis-exposed populations not only at the individual level but also at the community and system levels. A recent scoping review of international models of psychosocial preparedness identified community resilience and governance, task-sharing with non-specialist providers, and the integration of mental health and psychosocial support across the crisis cycle as recurrent priorities [40]. Viewed within such multi-level frameworks, the individual-level associations observed here represent only one component of a broader resilience architecture, and any preventive value is likely to depend on the alignment of individual, school, and community resources rather than on individual skill-building alone.

### Strengths and Limitations

This study has several strengths. It draws on a large, school-based, cluster-sampled sample; it assesses general and trauma-specific outcomes jointly rather than in isolation; and it is set in Türkiye, the country hosting the largest Syrian refugee population, where evidence on resilience remains scarce relative to high-income resettlement settings [2,26]. To our knowledge, it is also the first to quantify the unique contribution of resilience to both symptom domains after adjustment for demographic covariates in this population. Several limitations should also be noted. First, the cross-sectional design precludes causal inference, and the direction of association cannot be established; symptoms may also erode resilience over time. Second, trauma exposure was not measured and could not be included as a covariate, which is an important limitation given its central role in this population. Relatedly, the CRIES-8 was administered without anchoring to a specified index event, so intrusion and avoidance scores reflect symptoms in relation to heterogeneous, self-identified stressful experiences that may not have been war- or displacement-related for all participants; this constrains their interpretation as indicators of displacement-related post-traumatic stress specifically. Third, all measures were self-reported, raising the possibility of common-method variance, which may have inflated the observed associations, and administration was mixed-mode—predominantly paper-and-pencil in class, with 21 participants completing the survey online—which may introduce minor mode effects. Fourth, resilience was assessed with a brief, unidimensional scale that does not capture family- and community-level resources. In addition, although the CRIES-8 is widely used with conflict-affected youth, its reliability has been reported to be lower among Syrian adolescents specifically [15]. Moreover, the internal consistency of the BRS in the present sample was low (α = 0.40), which may reflect the reverse-keyed item structure of the scale and its performance in an Arabic- and Turkish-speaking adolescent sample; measurement error of this magnitude would tend to attenuate the observed associations and warrants cautious interpretation of the resilience effects. Fifth, the study did not include potentially important post-migration contextual variables, such as duration of stay in Türkiye, socioeconomic hardship, language proficiency, perceived discrimination, family separation, or access to psychosocial support. Moreover, although participants were recruited through a multistage cluster sampling procedure, school identifiers were not recorded in the dataset, and the potential clustering of observations at the school level could therefore not be evaluated. School-level intraclass correlations for adolescent psychosocial outcomes are generally modest (ICC = 0.01–0.07) in large multinational school surveys [41]; nevertheless, unmodelled clustering may have led to some underestimation of standard errors, and the corresponding significance tests should be interpreted with this in mind. Finally, only school-attending adolescents in a single city (Ankara) were sampled, limiting generalizability to out-of-school refugee youth and to Syrian adolescents in other regions of Türkiye. Data were collected between late 2019 and 2022, a period interrupted by pandemic-related school closures, when the Syrian population under temporary protection in Türkiye was close to its peak; substantial return movements have occurred since December 2024, and the composition of the remaining population may differ from that of the present sample, so the findings should be generalised to the current Syrian adolescent population in Türkiye with caution.

## 5. Conclusions

In a large sample of Syrian refugee adolescents in Türkiye, resilience was consistently and negatively associated with both general mental health problems and trauma-specific post-traumatic stress symptoms, after accounting for age and gender. Although the effects were modest, resilience represents a potentially modifiable correlate, and its role within school-based preventive programmes warrants further evaluation. Future research should employ longitudinal designs, measure trauma exposure, test whether resilience buffers the trauma–symptom association, and assess resources across multiple ecological levels.

## Figures and Tables

**Table 1:** Frequency statistics for the categorical variables (*N* = 501).

Variable	Level	*n*	%
Gender	Boys	319	63.67
	Girls	182	36.33
Grade	5	4	0.80
	6	8	1.60
	7	50	9.98
	8	40	7.98
	9	142	28.34
	10	108	21.56
	11	91	18.16
	12	58	11.58
Number of siblings	1	7	1.40
	2	17	3.39
	3	77	15.37
	4	132	26.35
	5	109	21.76
	6	66	13.17
	7+	93	18.56
Parental marital status	Married	412	82.24
	Separated	77	15.37
	At least one parent deceased	12	2.40

**Table 2:** Descriptive statistics and correlation coefficients among the study variables (*N* = 501).

Variable	M	SD	Skew	Kurt	1	2	3	4	5	6	7	8
1. Age	15.72	1.61	−0.44	−0.49	1							
2. Gender	1.36	0.48			−0.08	1						
3. Resilience	18.27	3.59	0.08	1.00	−0.10 *	0.12 **	1					
4. Depression	6.62	4.47	0.48	0.03	0.07	−0.13 **	−0.13 **	1				
5. Anxiety	6.35	4.23	0.37	−0.29	0.11 *	−0.15 **	−0.21 **	0.65 **	1			
6. Stress	6.69	4.54	0.36	−0.12	0.08	−0.13 **	−0.16 **	0.70 **	0.59 **	1		
7. Intrusion	9.01	3.58	0.04	−1.04	0.09 *	−0.16 **	−0.17 **	0.29 **	0.31 **	0.30 **	1	
8. Avoidance	9.13	3.53	−0.07	−1.02	0.11 *	−0.21 **	−0.18 **	0.22 **	0.26 **	0.26 **	0.75 **	1

Gender: 1 = boys, 2 = girls. * *p* < 0.05; ** *p* < 0.01.

**Table 3:** A summary of regression analysis.

Outcome	Predictor	B	SE	β	t	*p*	95% CI for B
Depression	Age	0.130	0.123	0.047	1.056	0.291	−0.112 to 0.372
	Gender	−1.056	0.414	−0.114	−2.552	0.011	−1.869 to −0.243
	Resilience	−0.135	0.056	−0.108	−2.425	0.016	−0.244 to −0.026
Anxiety	Age	0.223	0.114	0.085	1.951	0.052	−0.002 to 0.448
	Gender	−1.021	0.385	−0.116	−2.654	0.008	−1.776 to −0.265
	Resilience	−0.218	0.052	−0.186	−4.230	<0.001	−0.320 to −0.117
Stress	Age	0.164	0.124	0.058	1.316	0.189	−0.081 to 0.408
	Gender	−0.963	0.418	−0.102	−2.303	0.022	−1.785 to −0.142
	Resilience	−0.183	0.056	−0.145	−3.260	0.001	−0.293 to −0.073
Intrusion	Age	0.144	0.098	0.065	1.473	0.141	−0.048 to 0.336
	Gender	−0.993	0.329	−0.133	−3.019	0.003	−1.638 to −0.347
	Resilience	−0.142	0.044	−0.142	−3.208	0.001	−0.228 to −0.055
Avoidance	Age	0.178	0.095	0.081	1.870	0.062	−0.009 to 0.365
	Gender	−1.341	0.320	−0.183	−4.195	<0.001	−1.968 to −0.713
	Resilience	−0.143	0.043	−0.145	−3.323	0.001	−0.227 to −0.058

Gender: 1 = boys, 2 = girls. Model statistics: depression, R^2^ = 0.032, adjusted R^2^ = 0.026, ΔR^2^ = 0.011; anxiety, R^2^ = 0.065, adjusted R^2^ = 0.060, ΔR^2^ = 0.034; stress, R^2^ = 0.041, adjusted R^2^ = 0.035, ΔR^2^ = 0.021; intrusion, R^2^ = 0.050, adjusted R^2^ = 0.044, ΔR^2^ = 0.020; avoidance, R^2^ = 0.073, adjusted R^2^ = 0.067, ΔR^2^ = 0.021.

## Data Availability

The datasets generated and/or analysed during the current study are not publicly available but are available from the corresponding author upon reasonable request. The dataset collected for this study involves data from Syrian Refugee Adolescents in Türkiye. In accordance with our institutional ethics approval and the informed consent agreements signed by participants’ guardians, we are not permitted to publicly share raw data, as doing so could compromise participant confidentiality and privacy, even after de-identification, given the sensitivity of data involving minors.

## References

[1] UNHCR (2025). Mid-Year Trends 2025.

[2] Scharpf F., Kaltenbach E., Nickerson A., Hecker T. (2021). A systematic review of socio-ecological factors contributing to risk and protection of the mental health of refugee children and adolescents. Clin. Psychol. Rev..

[3] Blackmore R., Gray K.M., Boyle J.A., Fazel M., Ranasinha S., Fitzgerald G., Misso M., Gibson-Helm M. (2020). Systematic review and meta-analysis: The prevalence of mental illness in child and adolescent refugees and asylum seekers. J. Am. Acad. Child Adolesc. Psychiatry.

[4] World Health Organization (2025). Adolescent Mental Health.

[5] Presidency of Migration Management, Republic of Türkiye Temporary Protection Statistics. https://www.goc.gov.tr/gecici-koruma5638.

[6] Görmez V., Kılıç H.N., Orengul A.C., Demir M.N., Demirlikan Ş., Demirbaş S., Babacan B., Kınık K., Semerci B. (2018). Psychopathology and associated risk factors among forcibly displaced Syrian children and adolescents. J. Immigr. Minor. Health.

[7] Kandemir H., Karataş H., Çeri V., Solmaz F., Kandemir S.B., Solmaz A. (2018). Prevalence of war-related adverse events, depression and anxiety among Syrian refugee children settled in Turkey. Eur. Child Adolesc. Psychiatry.

[8] Eruyar S., Maltby J., Vostanis P. (2020). How do Syrian refugee children in Turkey perceive relational factors in the context of their mental health?. Clin. Child Psychol. Psychiatry.

[9] Uysal B., Yanik M., Tastekne F., Tuzgen E., Altinisik E., Acarturk C. (2022). Psychological problems and resilience among Syrian adolescents exposed to war. Eur. J. Trauma Dissociation.

[10] Karadag M., Ogutlu H. (2021). Prevalence of psychiatric symptoms among refugee adolescents in Turkey: A controlled study. Braz. J. Psychiatry.

[11] Fazel M., Reed R.V., Panter-Brick C., Stein A. (2012). Mental health of displaced and refugee children resettled in high-income countries: Risk and protective factors. Lancet.

[12] Hinchey L.M., Alahmad R., Gorski K., Jenuwine M., Nugent N., Rosenberg D., Mohiddin T., Javanbakht A. (2025). Predictors of risk and resilience to psychopathology in refugee youth: A longitudinal study. Dev. Psychopathol..

[13] Scherer N., Hameed S., Acarturk C., Deniz G., Sheikhani A., Volkan S., Örücü A., Pivato I., Akıncı İ., Patterson A. (2020). Prevalence of common mental disorders among Syrian refugee children and adolescents in Sultanbeyli district, Istanbul: Results of a population-based survey. Epidemiol. Psychiatr. Sci..

[14] American Psychiatric Association (2022). Diagnostic and Statistical Manual of Mental Disorders.

[15] Kankaanpää R., Vänskä M., Opaas M., Spaas C., Derluyn I., Jervelund S.S., Skovdal M., Durbeej N., Osman F., De Haene L. (2024). Psychometric properties of the Children’s Revised Impact of Event Scale (CRIES-8) among refugee adolescents from Afghanistan, Syria, and Somalia. Eur. J. Psychotraumatol..

[16] Ehlers A., Clark D.M. (2000). A cognitive model of posttraumatic stress disorder. Behav. Res. Ther..

[17] Aalto S., Punamäki R.-L., Vänskä M., Kankaanpää R., Turunen T., Lahtinen O., Derluyn I., Spaas C., De Haene L., Smith Jervelund S. (2025). Patterns of mental health problems and resilience among immigrant and refugee adolescents: A latent profile analysis. Eur. J. Psychotraumatol..

[18] Smith B.W., Dalen J., Wiggins K., Tooley E., Christopher P., Bernard J. (2008). The Brief Resilience Scale: Assessing the ability to bounce back. Int. J. Behav. Med..

[19] Southwick S.M., Bonanno G.A., Masten A.S., Panter-Brick C., Yehuda R. (2014). Resilience definitions, theory, and challenges: Interdisciplinary perspectives. Eur. J. Psychotraumatol..

[20] Panter-Brick C., Hadfield K., Dajani R., Eggerman M., Ager A., Ungar M. (2018). Resilience in context: A brief and culturally grounded measure for Syrian refugee and Jordanian host-community adolescents. Child Dev..

[21] Ungar M., Ghazinour M., Richter J. (2013). Annual research review: What is resilience within the social ecology of human development?. J. Child Psychol. Psychiatry.

[22] Gonçalves R., Marques M.J., Sibeoni J., Dias S. (2026). Can we protect refugee children from mental health problems?—A scoping review of modifiable factors to inform preventive interventions. BMC Med..

[23] Marley C., Mauki B. (2019). Resilience and protective factors among refugee children post-migration to high-income countries: A systematic review. Eur. J. Public Health.

[24] Thompson N.J., Fiorillo D., Rothbaum B.O., Ressler K.J., Michopoulos V. (2018). Coping strategies as mediators in relation to resilience and posttraumatic stress disorder. J. Affect. Disord..

[25] Dehnel R., Dalky H., Sudarsan S., Al-Delaimy W.K. (2022). Resilience and mental health among Syrian refugee children in Jordan. J. Immigr. Minor. Health.

[26] Popham C.M., McEwen F.S., Karam E., Fayyad J., Karam G., Saab D., Moghames P., Pluess M. (2023). Predictors of psychological risk and resilience among Syrian refugee children. J. Child Psychol. Psychiatry.

[27] Mattelin E., Paidar K., Söderlind N., Fröberg F., Korhonen L. (2024). A systematic review of studies on resilience and risk and protective factors for health among refugee children in Nordic countries. Eur. Child Adolesc. Psychiatry.

[28] Özaslan A., Yildirim M., Guney E., İlhan M.N., Vostanis P. (2024). Mental health problems and help-seeking behaviours of Syrian refugee adolescents: Mediating role of self-stigma. Psychol. Med..

[29] Sarıçam H. (2018). The psychometric properties of the Turkish version of the Depression Anxiety Stress Scale-21 (DASS-21) in community and clinical samples. J. Cogn.-Behav. Psychother. Res..

[30] Ali A.M., Ahmed A., Sharaf A., Kawakami N., Abdeldayem S.M., Green J. (2017). The Arabic version of the Depression Anxiety Stress Scale-21: Cumulative scaling and discriminant-validation testing. Asian J. Psychiatry.

[31] Doğan T. (2015). Adaptation of the Brief Resilience Scale into Turkish: A validity and reliability study. J. Happiness Well-Being.

[32] Younes M., Alzahrani M.R. (2018). Could resilience and flourishing be mediators in the relationship between mindfulness and life satisfaction for Saudi college students? A psychometric and exploratory study. J. Educ. Psychol. Stud..

[33] Perrin S., Meiser-Stedman R., Smith P. (2005). The Children’s Revised Impact of Event Scale (CRIES): Validity as a screening instrument for PTSD. Behav. Cogn. Psychother..

[34] Iz M., Ceri V., Layik M.E., Ay F.B. (2019). Prevalence of post-traumatic stress disorder following unintentional injuries in children. East. J. Med..

[35] Gravetter F.J., Forzano L.B. (2012). Research Methods for the Behavioral Sciences.

[36] Aldabbour B., Abuabada A., Alnaqa A., Alharazin A.N., Zoghbour M.S., Awadallah A.O., Awd M.N., Elkhaldy Y.M., Shehada M.T., Shehada M.A. (2026). Mechanisms of risk and resilience among war-affected adolescents in Gaza: The mediating role of posttraumatic stress and the moderating role of prosocial behavior. Eur. Child Adolesc. Psychiatry.

[37] Podsakoff P.M., MacKenzie S.B., Lee J.Y., Podsakoff N.P. (2003). Common method biases in behavioral research: A critical review of the literature and recommended remedies. J. Appl. Psychol..

[38] Sengun Filiz E., Patel R., Hodes M. (2026). Systematic review: Child and adolescent refugees and asylum-seekers’ contact with specialist mental health services. J. Am. Acad. Child Adolesc. Psychiatry.

[39] Bürgin D., Anagnostopoulos D., Vitiello B., Sukale T., Schmid M., Fegert J.M., Board and Policy Division of ESCAP (2022). Impact of war and forced displacement on children’s mental health—Multilevel, needs-oriented, and trauma-informed approaches. Eur. Child Adolesc. Psychiatry.

[40] Barlattani T., Trianni A., Bologna A., Trebbi E., Terrone G., Rossi R., Rossi A., Pacitti F. (2026). Psychosocial preparedness for disasters: A scoping review of international models and public health priorities. Curr. Psychiatry Rep..

[41] Shackleton N., Hale D., Bonell C., Viner R.M. (2016). Intraclass correlation values for adolescent health outcomes in secondary schools in 21 European countries. SSM Popul. Health.

